# Progress of Diseases of the Throat and Nose

**Published:** 1902-01-11

**Authors:** 


### PROGRESS OF DISEASES OF THE THROAT AND NOSE.

(*Continued from page* 238.)

**Diphtheria.**—Jobson Horne [7] has done good work in demonstrating that the ventricle of Morgagni very frequently harbours tubercle bacilli, and his further researches have shown that these recesses are not seldom the receptacle for diphtheria bacilli and may indeed be the only situation of the false membrane, which is then wholly concealed from inspection during life. He reports a case in point where a post-mortem examination alone revealed the membrane in the ventricles. The recent report, by Prof. Sims Woodhead,[8] on the bacteriological diagnosis of diphtheria in connection with cases admitted to the hospitals of the Metropolitan Asylums Board, are most valuable and interesting reading. The following digest of a part of the report is taken from the *British Medical Journal*:—*Presence of Diphtheria Bacilli at Time of Discharge.*—Out of 12,172 cases, diphtheria bacilli were found in 4,052 or 33·3 per cent. of the whole. "There can be no doubt," remarks the Professor, "that in a certain proportion of cases the patients when discharged had in their throats diphtheria bacilli which, under favourable conditions, might be transmitted from the patient to those with whom he or she afterwards might come in contact. I am strongly of the opinion that more attention should be paid to the continued isolation of diphtheria patients in whose throats even slightly virulent diphtheria bacilli still remain." *Failure to find diphtheria bacilli at the first examination* occurred in 607 cases in which they were discovered subsequently. *The persistence of diphtheria bacilli* was shown by repeated bacteriological examination of certain cases. In two cases diphtheria bacilli were found at the end of 200 days, and in 79 cases they persisted beyond 100 days from the day when they were first found in the throat. *Mixed infections* are shown by the statistics to be always more fatal than are simple diphtheria intoxications; but contrary to what is usually stated, the staphylococcus reinforcing the diphtheria bacillus appeared to be associated with a more fatal form of the disease than when a mixture of a streptococcus and the diphtheria bacillus was found. This is particularly true when the shorter and irregular forms of the diphtheria bacillus were present. *The true significance of the short bacillus* is open to doubt. The statistics, as Professor Woodhead states, indicate that it is really a pseudo-form, or has such slight virulence that it usually, but not always, gives rise to a very mild attack of the disease. *Fatality in Relation to Form of Bacillus.*—Cases certified as having diphtheria, but showing that form alone, had a fatality of only 3·3 per cent. During the two years 1895-6 the cases showing pure cultures of long diphtheria bacilli had a fatality of 18·9 per cent. The fatality among cases showing mixed diphtheria bacilli and streptococci was 20·5, or, omitting the cases in which only short forms were found, 22·3 per cent. Diphtheria bacilli *plus* staphylococci give a percentage fatality of 25·4, or omitting the cases with short bacilli, of 27·3 per cent. *Scarlet Fever complicated with Diphtheria.*—Among these cases, numbering 1,745, the fatality was only 13·1 per cent., as compared with 61·9 per cent. in the pre-antitoxin period 1890-94. *Cases certified as Diphtheria: No Diphtheria Bacilli Found.*—This occurred in 26·5 per cent. of the whole number of cases examined in the laboratory during 1895-6. Allowing for errors of observation, it is clear that in over 20 per cent. (or about 3,000) of the cases notified as suffering from diphtheria and admitted into the various hospitals, no bacteriological evidence of this disease appeared. The fatality among these cases in the aggregate was 9·3 per cent. lower than that for cases in which the diphtheria bacilli were found, but too high for non-diphtherial sore throat. It is clear that these cases were a mixture in doubtful proportion of sore throats other than diphtheria and diphtheria in which the cultural experiment failed.

*The bacteriological examination of scarlet fever* (altogether 1,275 cases) in which no diphtheria bacilli were present brought out some interesting results. When only staphylococci were demonstrated the fatality was as high as 17·7 per cent., when only streptococci 9·1 per cent. For all these cases the fatality was 12·8 per cent., as compared with 13·1 per cent. for cases mixed with diphtheria.

As regards treatment, the necessity of increasing the dose of antitoxin as compared with recent dosage in most cases of diphtheria has been emphasised by several writers. McCollum[9] urges that while in some cases 4,000 units will be enough, in others 60,000 or 70,000 units will be required. For patients in the early stage 4,000 to 6,000 units will be sufficient, but with severe infections, or infections of long standing, doses of 10,000 units should be given repeatedly until the desired result is obtained.

Kirton[10] refers to certain cases of diphtheria in children in which feeding by the mouth is impossible or inadvisable. Chief amongst these are the following:—

(1) Inability to swallow owing to pain or faucial swelling; (2) some cases of regurgitation, which will be referred to later; (3) entrance of food into the larynx in spite of slow and careful feeding indicated by the patient coughing when fed; (4) in certain cases, owing to struggling, the time taken and the exhaustion consequent on mouth feeding; and (5) continued vomiting—(*a*) vomiting may be present almost from the onset of diphtheria; (*b*) early vomiting, commencing about the middle or end of the second week; and (*c*) late vomiting, which occurs after some weeks of the disease. Generally speaking, vomiting which begins early is of graver import than that which starts late in the disease. It is well to remember that cases occur in which patients can be fed with a nasal tube without vomiting, although they vomit when fed by the mouth.

In some this is probably due to distaste for their nourishment. He finds that children do best with milk by the mouth, diluted with barley water, or in such form as Benger's or Mellin's food. Milk should generally be sweetened. All nourishment should be measured, and this specially so in severe cases. Children with regurgitation should be fed very slowly, and on no account should they be allowed to feed themselves. When regurgitation persists even with slow feeding, all liquids should be thickened. Patients who vomit, even with peptonised milk, should be fed by some other method than by the mouth.

In cases of *severe diphtheria*, in which vomiting ultimately supervenes, the period previously to this may be quite free from vomiting. In such cases the milk should be supplemented by beef tea, raw meat juice, eggs beaten up and mixed with a little brandy and milk—a good mixture being one egg, a little powdered sugar, half an ounce of brandy, and milk to two ounces. This may be given in small portions over 12 or 24 hours. The nourishment should be given in small quantities at frequent intervals. Great care must be taken to satisfy oneself that the nourishment taken is absorbed. These are cases in which one anticipates early or late vomiting, when the feeding will become more and more difficult; therefore, if possible, they should be fed with due care previously to the onset of vomiting.

The following conditions render nasal feeding advisable: (1) inability to swallow from regurgitation, paralysis of muscles of deglutition, etc.; (2) coughing on feeding, due to entrance of food into the larynx, and this especially in tracheotomy cases; (3) cases which vomit when fed by the mouth, but do not when fed through the nose; (4) cases exhausted by mouth feeding, but not when nasal-fed (amongst these must be numbered cases in which mouth-feeding takes a very long time); and (5) moral effect. These suggestions apply to children who refuse food though well able to swallow. One nasal feed is often sufficient.

Generally the patient should be fed every four hours, but this must to a certain extent depend on the individual case. Milk, if necessary peptonised or as Benger's food, should be the chief ingredient. Raw meat juice, eggs, cream, brandy (if ordered), or medicines may be added to the diet. The quantity in each meal must vary with the age, size, etc., of the patient. About four ounces is often as much as a child of from three to five years old will be able to retain and to absorb.

*Rectal Alimentation.*—This should be resorted to when feeding by the mouth or nose fails or is insufficient. The following are the chief conditions where it is required: (1) Vomiting; (2) Great difficulty in passing the nasal tube from various causes; (3) When nasal feeding acts harmfully on the patient, owing to fright, struggling, or other cause; and (4) Epistaxis caused by passage of the nasal tube.

Rectal feeding should be performed by means of a funnel and soft rubber tube, passed as high up the rectum as possible, and the feed allowed to flow in by force of gravity. A ball syringe should not be used, for it is impossible to gauge accurately the amount of pressure used; experience also shows that nutrients given this way are not so well retained. The feeds should be given warm about every four hours. The feeds should have as their basis peptonised milk, and often this is their only constituent. Raw meat juice, white of eggs, and occasionally yelks of eggs are added.

[7] Jour. of Laryng., June, 1901. [8] Brit. Med. Jour., Digest in, Sept. 21, 1901. [9] Boston Med. and Surg. Jour., Vol. CXLIII., No. 25. [10] Lancet, June 15, 1901.

